# Hirsutism as the initial presentation of malignant ovarian Leydig cell tumor: A case report

**DOI:** 10.1002/ccr3.7915

**Published:** 2023-10-18

**Authors:** Hasan Arafat, Marah Khaldy, Ahmad Abu Munshar, Amer Zughayyer

**Affiliations:** ^1^ Cancer Care Center Augusta Victoria Hospital Jerusalem Palestine

**Keywords:** chemotherapy, irregular menstrual cycle, ovarian neoplasms, steroid cell tumors

## Abstract

Ovarian steroid cell tumors are a rare subtype of sex‐cord stromal cell tumors. Overall, these tumors make <0.1% of all ovarian tumors. These neoplasms can be divided according to the cell of origin into stromal luteomas, Leydig cell tumors, and steroid cell tumors not otherwise specified. These tumors can be benign, malignant, or borderline, with variable presentation. We report a case of 24‐year‐old virgin female who was referred to our hospital after being diagnosed with steroid cell tumor‐not otherwise specified. Prior to her admission, the patient had been treated unsuccessfully with oral contraceptive pills due to male‐pattern facial hair growth, abdominal cramps, and irregular menstrual cycle. Lack of improvement warranted further investigations. Hormonal studies showed an elevated total testosterone, dehydroepiandrosterone sulfate, and morning fasting cortisol. Ultrasonography and computed tomography confirmed the presence of a large pelvic mass with mixed solid and cystic component. Therefore, unilateral salpingo‐oophorectomy was performed. Pathological and immunohistochemical examination suggested the presence of a large ovarian steroid cell tumor‐not otherwise specified with malignant behavior. The patient did not receive adjuvant therapy and developed metastatic disease. She received four cycles of BEP protocol with no improvement, so she was referred to our center to continue oncological management. Case revision confirmed the presence of steroid cell tumor, but of a different subtype: Leydig cell. She received six cycles of carboplatin‐paclitaxel, but her assessment showed disease progression. We report this case with review of literature regarding the appropriate approach to these rare tumors. Although rare, ovarian steroid cell tumors should be included in the differential diagnosis of virilization in young females, especially those refractory to hormonal therapy. In our study, we aimed to present the first reported Palestinian case, which highlights the importance of detailed morphological examination in addition to the difficulties encountered to reach a proper diagnosis. We also provided a review of the existing literature regarding chemotherapeutic lines used in such cases and the response to each.

## Graphical abstract

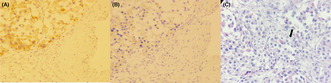

## 1 BACKGROUND

Ovarian steroid cell tumors (SCT) constitute a rare subtype of sex cord stromal cell tumors, they comprise <0.1% of all ovarian tumors. These neoplasm can be classified according to their cell of origin into three classes: stromal luteoma, Leydig cell tumor (LCT), and steroid cell tumor‐not otherwise specified (SCT‐NOS).^1^ Their behavior can be benign, malignant, or borderline, with nearly a quarter presenting with no hormonal disturbances.^2^ Due to their rarity, identifying them as the underlying cause of female hirsutism and virilization can be challenging.^3^ The diagnosis of SCT can be suggested by a combination of imaging and biochemical tests, but histopathology is essential for the correct diagnosis and grading.

We present herein a case of a Palestinian young female patient diagnosed with steroid cell tumor, Leydig cell subtype of the ovary presenting with hirsutism, posing a difficulty in diagnosis due to the accompanying biochemical changes as well as histopathological similarities. This case is the first reported Palestinian case to our knowledge. This article was previously posted to the Research Square preprint server on April 13, 2023.

## 2 CASE PRESENTATION

A 24‐year‐old unmarried Palestinian female from Palestine, with an unremarkable past medical and past surgical history, presented to obstetrics and gynecology (OBGYN) clinic in December 2021 with a history of 4 months of irregular menstrual cycle and prolonged bleeding associated with diffuse abdominal cramps, with each episode lasting for 2 weeks. The patient had menarche at age 12, her cycles were regular with 3 days of bleeding each month. She also mentioned increased hair growth over the upper lip, armpits, and between breasts, corresponding to a Ferriman‐Gallwey score of 11. Her reports do not mention abnormal vitals, moon face, abdominal obesity, abdominal striae, or buffalo hump (negative for cushingoid features). She was treated with simple analgesics and oral contraceptive pills (OCPs), composed of 0.03 mg of ethinylestradiol combined with 3 mg of drospirenone daily for 21 and 7 days break for 3 months, but she showed no improvement. Hormonal profile tests were intriguing: total testosterone (TT) was high (2.40 ng/mL), but the level of dehydroepiandrosterone sulfate (DHEA‐S) was extremely high (over 1000 μg/mL, the machine could not specify a value beyond that level) with high fasting, morning cortisol (41.0 ng/mL). Prolactin, blood sugars, and all other blood parameters were within normal limits. Normal values can be found in Table 1 below. Abdominal ultrasound showed a 13 × 14 cm heterogenous lower abdominal mass, follow up computed tomography (CT) scan showed a large pelvic abdominal cystic mass with solid component, heterogenous in density, multilobulated, measuring 24 × 12 cm, displacing bowel loops peripherally, while displacing the uterus and bladder anteriorly. The case was discussed by a team of gynecologists and oncologists, who opted for right salpingo‐oopherectomy in March 2022. Grossly, the mass was described as being multilobulated, with the largest lobe measuring 20 × 30 cm. Its surface was shiny, yellowish, soft with fragile overlying tissue. On microscopic examination, the tissue showed steroid cell tumor of adrenocortical type, alternatively categorized as not otherwise specified, with large size necrosis, nuclear atypia and increased mitotic activity, all pointing to possible malignant behavior. The patient was staged as having stage IC2 disease, so she did not receive adjuvant chemotherapy as the tumor was completely resected. She was kept on regular follow‐up. On May 2022, the patient returned to oncology clinic complaining of recurred abdominal pain, new CT scan with IV contrast demonstrated recurrence of disease with the presence of two large, metastatic retroperitoneal lymph nodes with heterogenous enhancement, the largest of which measured 3.3 × 4 cm at the level of the third lumbar vertebral body and the smaller one measured 2.2 × 3 cm in the left para‐aortic area at the level of the second lumbar vertebral body. There was also bilateral parailiac small lymph node enlargement, suggesting recurrent, metastatic stage four disease.

**TABLE 1:** Hormonal profile before and after surgery.

	Before surgery	7 months after surgery	Normal values
Total testosterone	2.4	<0.025	0.1–1.2 ng/mL
Fasting morning cortisol	41.0	5.25	4.82–19.5 ng/mL
CA125	Not done	16.4	<35 U/mL
DHEA‐S	>1000	67.9	98.8–340.0 μg/mL
Prolactin	10.5	8.36	1.9–25.9 ng/mL
ACTH	Not done	39.15	9–52 pg/dL
FSH	<0.05	20.96	Follicular: 3.3–8.8 mIU/mL, midcycle: 5.4–21.5 mIU/mL, luteal: 1.6–8.7 mIU/mL
LH	0.03	17.34	Follicular & luteal: 5.0–15 mIU/mL. Midcycle: 14–60 mIU/mL

Abbreviations: ACTH, adrenocorticotropic hormone; DHEA‐S, dehydroepiandrosterone‐sulfate; FSH, follicle stimulating hormone; LH, luteinizing hormone.

The patient received four cycles of BEP protocol (Bleomycin, 30 mL on days two, nine and 16, with cisplatin, 20 mg/m^2^ on days one to five, and etoposide, 100 mg/m^2^) administered every 21 days intravenously (IV). Unfortunately, her first assessment for disease response on August 2022 showed an increase in the size of the previously mentioned metastatic lymph nodes, in addition to early omental infiltration, indicating disease progression (DP). The patient was referred to our specialized oncology center, the Augusta Victoria Hospital in Jerusalem.

On presentation, the patient was complaining of mild back pain but reported no abdominal pain. Her height and weight were 150 cm and 65 kg, respectively, corresponding to a body mass index of 28.9 kg/m^2^. Physical examination revealed the absence of any abdominal tenderness or palpable masses, no signs of hirsutism. The patient mentioned that her menstrual cycle had returned to normal following surgery. Hormonal workup showed low levels of TT (<0.025 ng/mL), normal fasting morning cortisol (5.25 ng/mL), low levels of DHEA‐S (67.9 μg/dL), estradiol (<5.0 pg/mL), and CA 125 (16.40 U/mL). CT scan was repeated, disclosing few suspicious abdominal wall lesions, the largest measuring 8 mm, with few retroperitoneal and right parailiac lymph nodes, in addition to omental implants at the splenic hilum and midabdomen. Confirming the presence of stage four metastatic cancer. Table 1 compares hormonal test results prior and after surgery.

Due to the rarity and complexity of the case, it was discussed in the weekly tumor board regarding possible second line and the feasibility of cytoreductive surgery. The discussing multidisciplinary team (including medical oncology, oncological surgery, pathology, and endocrinology) agreed on starting a new line chemotherapy, disfavoring surgical intervention at the current disease status. In addition, the pathologists suggested bringing paraffin blocks for histopathology case revision, as per the center policy.

A new line chemotherapy protocol was commenced, consisting of carboplatin, area under the curve (AUC) six, plus paclitaxel 175 mg/m^2^ IV every 21 days, in addition to goserelin 10.8 mg subcutaneously every 3 months as a means of fertility preservation. She was scheduled for assessment after six cycles. She brought paraffin blocks for case revision at the second cycle.

**FIGURE 1:**
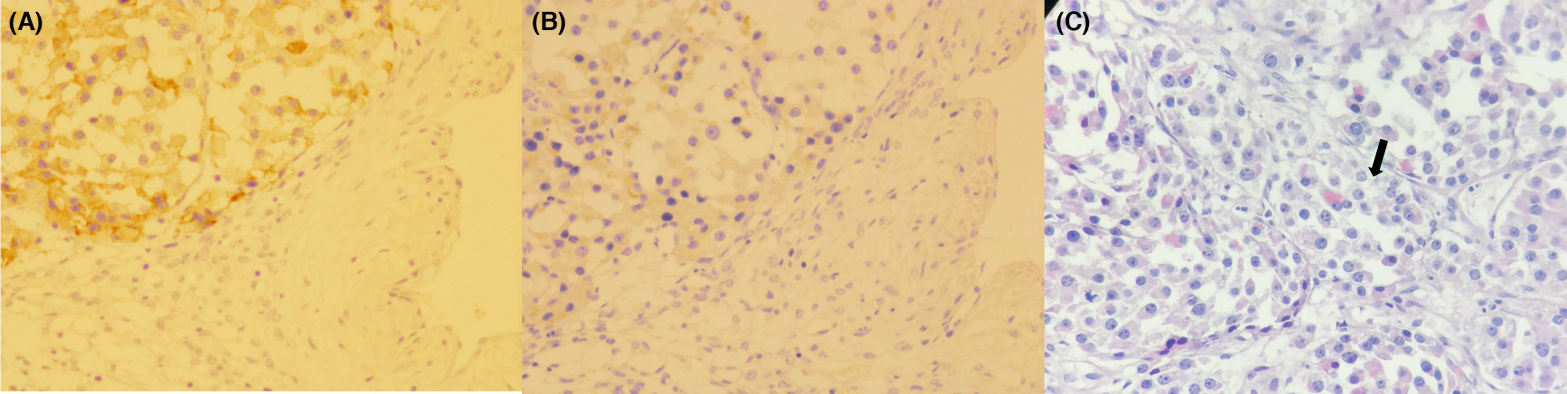
Immunohistochemistry of the neoplasm. Section A shows high‐positivity for inhibin, section B shows high positivity for melan‐A, and section C shows the crystals of Ranke, tip of the arrow.

Case revision confirmed the presence of ovarian steroid cell tumor, but contradicted the original report in that it inclined toward Leydig cell origin instead of NOS. Microscopic examination revealed moderate atypia, with increased mitotic activity and some areas of necrosis. Borders showed focal infiltration. Immunohistochemistry was positive for vimentin, CD99, with focal positivity for inhibin (Figure 1A), synaptophysin, NSE and melan‐A (Figure 1B). The cells were negative for pan‐CK, epithelial membrane antigen (EMA), S100, SALL4, PAX8, and AFP. Crystals of Ranke could also be observed (Figure 1C). The overall features were mostly inclined toward malignant behavior. Management was not changed and she continued on the same protocol.

The patient received a total of six cycles of carboplatin‐paclitaxel, then underwent chest‐abdomen‐pelvis CT for evaluation of disease response, results showed an increase in the perigastric lymph nodes, evidence of mild increase in the size and heterogenicity of the para‐aortic lymph nodes, with newly developed multiple omental deposits (supra‐ and infra‐umbilical). The presence of non‐specific inguinal lymphadenopathy was also noted. In conclusion, the patient was found to have DP. As the patient kept deteriorating on chemotherapy, the case was discussed again by our team of oncologists, it was concluded that the patient needed to be referred to a highly qualified center for cytoreductive surgery, to be followed by possible adjuvant chemotherapy with VAC protocol (vinblastine, doxorubicin, and cisplatin).

## 3 DISCUSSION

Ovarian steroid cell tumors represent <0.1% of all ovarian neoplasms, with Leydig cell subtype being one of the rarest subtypes. They are mostly found in postmenopausal women in their late fifties, although cases in younger individuals were also reported. Malignant behavior is rare in female individuals with a handful of reported cases, and these tumors are usually resistant the chemotherapy or radiation, with a high rate of recurrence after surgery.^4^ It is difficult to differentiate the different subtypes of ovarian steroid tumors.^5^ Our case describes a 24‐year‐old female presenting with hyperandrogenic symptoms. The patient was diagnosed with ovarian steroid tumor‐not otherwise specified initially, but after reviewing the tissue sample in our advanced oncology center, our pathologists were more in favor of Leydig cell subtype. The difficulty and challenges surrounding proper diagnosis represents a unique point to our case in addition to being the first case in Palestine.

Pre‐operatively, the diagnosis of SCT is suspected via clinical, biochemical, and imaging techniques. Patients with LCT often present with symptoms of abdominal distension and bloating.^6^ Steroid cell tumors are usually virilizing. Many of them may be endocrinologically inert or may be estrogenic. One quarter of these tumors exhibit malignant behavior.^7^ Leydig cell tumors usually present with rapidly progressive clinical manifestations of hyperandrogenism. Hypercortisolism is not an infrequent association of steroid cell tumors. However, clinical symptoms alone do not provide a reliable clue toward the source of hyperandrogenism. Hormonal studies constitute an important diagnostic tool for proper diagnosis.^8^, ^9^ Our patient's main complaints were male‐pattern hair growth, abdominal pain and distension, in addition to irregular menses. She was initially treated with OCPs. Lack of improvement prompted her physician to refer her for imaging, which pointed toward her actual diagnosis. She had high levels of morning cortisol, DHEA‐S and estradiol, but a normal level of CA 125, and total testosterone, which may be attributed to use of OCPs.

Leydig cell tumors are usually unilateral, small, only slightly larger than the normal ovary and are classically described as being difficult to characterize on radiological imaging. These tumors are isoechoic on ultrasound, however, careful assessment of the texture of the ovary might reveal an area of different echogenicity corresponding to the solid component of the tumor. When viewed on CT scan, these tumors are identified by their very low density, despite being isoattenuating when compared to the nearby uterus.^6^, ^10^ In our case, the mass was detected via abdominal ultrasound, CT scan confirmed its presence, differentiating it via its solid component.

Each of the subtypes of ovarian sex cord‐stromal tumors, including steroid cell tumors, can be in the differential diagnosis of each other's.^11^ Microscopically, steroid cell tumors pattern of proliferation is characterized by the presence of large polygonal cells with vacuolated cytoplasm and smaller cells with eosinophilic cytoplasm. The presence of crystals of Reinke is helpful to distinguish LCT from SCT‐NOS, the last is not accompanied by Leydig cell hyperplasia. The absence of spindle cells and fibromatous background is useful to distinguish SCT from luteinized thecoma.^12^ General immunohistochemistry observations might be useful in problematic cases; for example, CD99 is expressed in all sex cord stromal tumors except for SCT‐NOS and fibromas. Inhibin and calretinin are expressed less with fibromas. Primary ovarian carcinomas often display diffuse positivity for CK7 and EMA and negative for inhibin and calretinin. On the contrary, sex cord‐stromal tumors are usually negative for CK7 and EMA. While variabilities exist, the pattern of expression varies. For example, LCTs exhibit a focal pattern of expression for CK7 and EMA in contrast to carcinomas, which exhibit a diffuse pattern when positive. Chromogranin and synaptophysin in sex cord‐stromal tumors is either absent or focally positive.^13^

Proper staging is essential in these tumors, which is based on the International Federation of Gynecology and Obstetrics (Fédération Internationale de Gynécologie Obstétrique, FIGO) system, with most tumors being in stage I. Gold standard for diagnosis is total hysterectomy with bilateral oophorectomy. In women in child‐bearing age, unilateral salpingo‐oophorectomy is also an option in stage IA‐IC disease. In older women, as well as patients with advanced disease, total hysterectomy with bilateral salpingo‐oophorectomy, removal of the omentum and metastatic deposits is considered standard. Advanced cases should be followed up by either BEP protocol (bleomycin, etoposide, and cisplatin), VAC protocol (vinblastine, doxorubicin, and cisplatin) or carboplatin‐paclitaxel. Recurrent disease might necessitate recurrent surgery, radiotherapy, chemotherapy, or even hormonal therapy. Patients should be followed up appropriately following definitive therapy.^14^

Our patient underwent unilateral salpingo‐oophorectomy for tissue biopsy and therapy, it was preferred due to the patient young age as a means of fertility preserving. The patient did not receive adjuvant chemotherapy as she had stage IC2 disease. She developed metastatic disease within 3 months. She received four cycles of BEP protocol. Her first assessment showed DP. The patient was referred to our hospital, and the case was discussed in the tumor board by a multidisciplinary team. Our onco‐surgeons disfavored cytoreductive surgery, inclining more toward giving the patient preoperative chemotherapy then assessing for the feasibility of surgery afterwards. Six cycles of carboplatin AUC‐six with paclitaxel were given to the patient; however, the patient showed DP. Her case will be also discussed for the possibility of cytoreductive surgery in addition to new line VAC protocol, due to failure of two lines of chemotherapy.

## 4 CONCLUSION

Steroid ovarian cell tumors constitute a rare cause of virilization in women, with only a handful of cases reporting Leydig cell subtype. They should be considered in the differential diagnosis of women presenting with signs and manifestations of hormonal imbalance. Each subtype of these tumors has a slightly unique immune profile in terms of frequency and extent of expression. In this manuscript, we aimed to illustrate the first case of this tumor reported in Palestine, demonstrating the difficulties faced by clinicians in diagnosing this entity and the importance of histopathological examination and ancillary techniques in achieving proper diagnosis and differentiating the different subtypes. We also demonstrated the importance of multidisciplinary team discussion in approaching rare diseases with scarce literature.

## AUTHOR CONTRIBUTIONS

**Hasan Arafat:** Conceptualization; data curation; formal analysis; project administration; validation; writing – original draft; writing – review and editing. **Marah Khaldy:** Writing – original draft. **Ahmad Abu Munshar:** Data curation. **Amer Zughayyer:** Conceptualization; supervision.

## FUNDING INFORMATION

The authors received no financial support for the research, authorship, and publication of this article.

## CONFLICT OF INTEREST

The authors declare that they have no conflict of interest.

## ETHICAL APPROVAL AND CONSENT TO PARTICIPATE

Not applicable.

## CONSENT

Written informed consent was obtained from the patient to publish this report in accordance with the journal's patient consent policy. A copy of the written consent is available for review by the Editor‐in‐Chief of this journal.

## Data Availability

The data that support the findings of this study are available from the corresponding author, HA, upon reasonable request. This manuscript is available as a preprint at Research Square, link to preprint: https://doi.org/10.21203/rs.3.rs‐2800156/v2.
